# The Impact of the Share 35 Policy on Racial and Ethnic Disparities in Access to Liver Transplantation for Patients with End Stage Liver Disease in the United States: An Analysis from UNOS Database

**DOI:** 10.1186/s12939-017-0552-8

**Published:** 2017-03-24

**Authors:** Yefei Zhang

**Affiliations:** Department of Biostatistics, School of Public Health, University of Texas Health Science Center at Houston, 1200 Pressler Street, RAS-E803f, Houston, TX 77030 USA

**Keywords:** Racial and ethnic disparities, Liver transplantation, Share 35 policy, United Network for Organ Sharing

## Abstract

**Background:**

The Share 35 policy was instituted in June 2013 by the United Network for Organ Sharing (UNOS) in order to reduce death on liver transplant waiting list. The effect of this policy on racial and ethnic disparities in access to liver transplantation has not been examined.

**Methods:**

A total of 14,585 adult patients registered for liver transplantation between 2012 and 2015 were identified from UNOS database. Logistic and proportional hazards models were used to model the effects of race and ethnicity on access to liver transplantation. Stratification on pre- and post-Share 35 periods was performed to compare the first 18 months of Share 35 policy to an equivalent time period before.

**Results:**

Comparison of the pre- and post-Share 35 periods showed significantly decreased time on waiting list and increased numbers of minorities having access to liver transplantation. Hispanic recipients still experienced significantly longer waiting time (HR: 0.69, 95% CI: 0.53–0.88) before they received liver transplantation after Share 35 policy took effect.

**Conclusion:**

The Share 35 policy did not lead to improved access to liver transplantation among minorities but eliminated the previously observed racial and ethnic disparities in transplant rates as well as shortened the waiting time.

## Background

According to the U.S. Census Bureau, the United States is projected to become more racially and ethnically diverse in the coming years [1]. Although the non-Hispanic White is currently the majority group accounting for 62.2% of the nation’s total population, the majority and minorities (any group other than non-Hispanic White alone) crossover is estimated to occur in 2044 [1]. In addition, by 2060, 56% of all Americans are projected to belong to a minority group [1]. The African American (AA) population is expected to encompass 14.3% of the U.S. population, while Hispanics will account for nearly 30% of the U.S. population [1].

In the past decades, AAs have experienced higher mortality rates from end-stage liver disease (ESLD) than non-Hispanic whites, and this pattern has also been observed more recently in Hispanics [2]. Data from Centers for Disease Control and Prevention identified that mortality from ESLD in Hispanics in the U.S. was almost 50% higher than in non-Hispanic whites (13.7 per 100,000 in Hispanics vs 9.2 in non-Hispanic whites) [3]. As liver transplantation being the only curative option for patients with ESLD at present, more than 14,000 people in the United States are currently waiting for liver transplantation (7.7% being AAs and 17.8% Hispanics) [4]. However, patients’ access to liver transplantation has been limited because of the widening gap between the increasing number of transplantation candidates on the waiting list and the number of available livers [5]. In addition, it has been reported that AAs had significantly lower transplant rates both in the eras prior and posterior to the adoption of the Model for End-Stage Liver Disease (MELD) score in 2002, a numeric scale ranging from 6 (less ill) to 40 (gravely ill) used for liver transplantation candidates age 12 and older to evaluate how urgently he or she needs a liver transplantation within the next 3 months [6]. Recent data also observed higher risks for developing alcohol-related liver disease and higher MELD scores in minority populations while waiting on the list, particularly among AAs and Hispanics [2, 3, 7–9].

On June 18, 2013, the Share 35 policy was instituted by the United Network for Organ Sharing (UNOS), a private, non-profit organization that manages the nation’s organ transplant system under contract with the federal government [10]. The Share 35 policy mandates that regional sharing of livers (livers distributed to the 11 UNOS allocation regions in the U.S.) for patients with a MELD score of ≥ 35 is prioritized over local sharing (livers distributed to candidates located in the same region as the donor) to patients with a MELD score of < 35 in order to reduce the waiting list mortality. Previous studies have evaluated race and ethnic disparities on access to liver transplantation but failed to adjust for geographic factors that may affect the availability of livers from deceased donors, and others failed to take the effect of Share 35 policy into account [11–14].

Therefore, given the rapid diversification of the U.S. population, the growing burden of liver disease in minority communities, as well as the implementation of Share 35 policy, the objective of the paper was to address the impact of the Share 35 policy on racial and ethnic differences in access to liver transplantation by comparing the first 18 months of the initiation of the policy to an equivalent time period before, using the UNOS database, while accounting for patient differences and geographic factors.

## Methods

### Data source

Data were collected by each transplant center and transmitted to UNOS. Detailed descriptions of the UNOS registry have been published elsewhere [15]. Briefly, the UNOS registry records and documents any change in standard demographic, clinical, and laboratory information available at the time of listing, during transplantation, and post-transplantation, as well as information on the donors. The UNOS data set contains one record per transplantation event.

The committee for the Protection of Human Subjects at the University of Texas Health Science Center at Houston approved this study.

### Study population

**Fig. 1:**
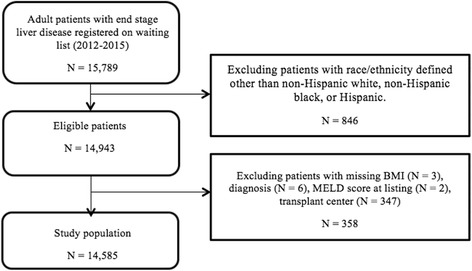
Flowchart for inclusion and exclusion of study population

A total of 15,789 liver transplant candidates with ESLD, 18 years of age and older, with an initial data of registration for deceased donor liver transplantation between January 1st, 2012 and March 31st, 2015 were identified from the wait list and liver file of the UNOS database. Only candidates with race/ethnicity defined as non-Hispanic white, non-Hispanic AA, and Hispanic were selected (*n* = 14,943). Patients with other races, more than one races, unknown race were not included (*n* = 646, 4%) due to small numbers. Candidates were then excluded for the following reasons: missing Body Mass Index (BMI) (*n* = 3), missing diagnosis (*n* = 6), unknown MELD score at listing (*n* = 2), and unknown transplant center (*n* = 347). After all exclusions, a total of 14,585 patients were available for analysis (Fig. 1).

### Study variables

The primary outcome, the access to liver transplantation, was measured by 1): the receipt of liver transplantation, and 2) the total time on the waiting list before the receipt of liver transplantation in days.

The exposure variable of primary interest was race/ethnicity as reported in UNOS records, classified as non-Hispanic white, non-Hispanic AA, and Hispanic. Other demographic and clinical variables included age at listing in years, gender (male or female), BMI measured in units of kg/m^2^, diagnosis, MELD score at listing, health insurance status, median household income in current dollars, geographical region, and transplant center.

Follow-up for patients began when they were initially added to the waiting list. Patients were then followed until the earliest of liver transplantation, death, the granting of a MELD exception score, or the end of the study. Patients who received a liver transplantation, alive, or lost to follow-up were censored at the date of transplantation or last follow-up.

### Statistical analysis

Frequencies and percentages were calculated for the baseline demographic and clinical characteristics. Chi-square tests and ANOVA tests were used to compare the characteristics among three racial and ethnical groups as well as between pre-Share 35 and post-Share 35 periods. Logistic regression models were used to model the effects of race and ethnicity on the receipt of liver transplantation adjusting for all other demographic and clinical characteristics. Cox proportional hazards models were used to model the effects of race and ethnicity on the total waiting time on the list before the receipt of liver transplantation in days adjusting for all other demographic and clinical characteristics. To account for the impact of geographic factors, regions or transplant centers were incorporated as clusters in all regression models by a marginal approach with a working independence assumption. Stratifications between pre-Share 35 period and post-Share 35 period were also performed. All statistical analyses were performed with SAS version 9.4 (SAS Institute Inc., Cary, NC). *P*-values less than 0.05 were considered statistically significant.

## Results

The baseline demographic and clinical characteristics of the 14,585 patients were displayed in Table 1. Compared to the pre-Share 35 cohort, racial and ethnic subgroups in the post-Share 35 cohort were similar with respect to the following factors: age at wait-listing, gender, BMI at listing, median household income, and health insurance status. The mean time on waiting list decreased significantly from 67 days to 32 days for AAs and from 107 days to 40 days for Hispanics. Compared to pre-Share 35 period, there were higher proportions of both AAs (87.0%) and Hispanics (86.9%) in the post-Share 35 period receiving liver transplantation. AA patients had lower proportions in being on the waitlist (3.0%), being too sick to receive transplantation (7.2%), having insurance issues (1.8%) and withdrawn from waitlist (0.5%). Hispanic patients experienced reduced proportions in being too sick to receive transplantation (7.0%) and having insurance issues (1.1%). There were significantly less patients with MELD score at listing below 35 and more patients with MELD score greater than 35 registered on the waiting list for all the three racial and ethnic subgroups after the implementation of the Share 35 policy.

**Table 1:** Baseline demographic and clinical characteristics of waiting list candidates in the entire cohort by race and ethnicity: Pre-Share 35 vs. Post-Share 35 time periods, 2012–2015

	Pre-Share 35 (*n* = 7450)			Post-Share 35 (*n* = 7135)		
	White (*n* = 5633)	AA (*n* = 798)	Hispanic (*n* = 1019)	White (*n* = 5351)	AA (*n* = 793)	Hispanic (*n* = 991)
Age at listing (years), mean (SD)	57.0 (9.9)	57.0 (11.4)	56.0 (10.2)	58.0 (10.5)	57.0 (12.9)	56.0 (11.3)
Gender, n (%)						
Male	3813 (67.7)	485 (60.8)	679 (66.6)	3603 (67.3)	462 (58.3)	648 (65.4)
Female	1820 (32.3)	313 (39.2)	340 (33.4)	1748 (32.7)	331 (41.7)	343 (34.6)
BMI, mean (SD)	27.9 (5.6)	27.5 (6.0)	27.7 (5.3)	27.9 (5.9)	27.5 (6.6)	28.3 (5.9)
Median household income^a^	54269	43294	47143	54249	44723	46394
Time on waiting list (days), mean (SD)	92.0 (200.5)	67.0 (202.3)	107.0 (211.5)	53.0 (116.9)	32.0 (109.8)	40.0 (124.8)
Waiting list outcome, n (%)						
Transplanted	4571 (81.2)	659 (82.6)	868 (85.2)	4550 (85.0)	690 (87.0)	861 (86.9)
Still waiting	233 (4.1)	26 (3.3)	36 (3.5)	163 (3.1)	24 (3.0)	35 (3.5)
Temporarily too sick	614 (10.9)	83 (10.4)	90 (8.8)	482 (9.0)	57 (7.2)	69 (7.0)
Insurance issues	88 (1.6)	18 (2.3)	13 (1.3)	69 (1.3)	14 (1.8)	11 (1.1)
Medical non-compliance	52 (0.9)	1 (0.1)	6 (0.6)	22 (0.4)	3 (0.4)	6 (0.6)
Candidate withdrawn	71 (1.3)	10 (1.3)	4 (0.4)	62 (1.2)	4 (0.5)	7 (0.7)
Candidate cannot be contacted	4 (0.1)	1 (0.1)	2 (0.2)	3 (0.1)	1 (0.1)	2 (0.2)
MELD at listing, n (%)						
< 35	5158 (91.6)	697 (87.3)	886 (87.0)	4619 (86.3)	602 (75.9)	796 (80.3)
≥ 35	475 (8.4)	101 (12.7)	133 (13.0)	732 (13.7)	191 (24.1)	195 (19.7)
Health insurance status, n (%)						
Private insurance	3301 (58.6)	382 (47.9)	454 (44.6)	3100 (57.9)	371 (46.8)	437 (44.1)
Public insurance	2292 (40.7)	405 (50.8)	552 (54.2)	2157 (40.3)	407 (51.3)	529 (53.4)
Self	21 (0.4)	7 (0.9)	5 (0.5)	19 (0.4)	1 (0.1)	4 (0.4)
Other	12 (0.2)	2 (0.3)	5 (0.5)	18 (0.3)	3 (0.4)	1 (0.1)
Unknown	7 (0.1)	2 (0.3)	3 (0.3)	57 (1.1)	11 (1.4)	20 (2.0)

*AA* African American, *BMI* body mass index, *MELD* model for end-stage liver disease

^a^Source: U.S. Census Bureau. Census tract level income in 2016 dollars

Table 2 displayed the risk-adjusted odds ratios for liver transplant rates among patients registered on waiting list for both pre- and post-Share 35 periods. AA patients were 11% more likely to receive liver transplantation after the Share 35 policy took effect (OR: 1.11, 95% CI: 0.89–1.40), but the association was non-significant compared to white patients. In contrast to AAs, Hispanics had a significantly higher liver transplant rate (1.34, 1.11–1.63) versus whites during the pre-Share 35 period. However, they did not have significantly different transplant rates in comparison to whites (1.15, 0.94–1.41) during post-Share 35 period.

**Table 2:** Logistic regression results for the receipt of liver transplantation among patients registered on waiting list: Pre-Share 35 vs. Post-Share 35 time periods, 2012–2015

	Pre-Share 35 (*n* = 7450)	Post-Share 35 (*n* = 7476)
	Odds Ratio (95% Confidence Interval)^a^	Odds Ratio (95% Confidence Interval)^a^
White	1.00 (reference)	1.00 (reference)
AA	1.09 (0.89–1.33)	1.11 (0.89–1.40)
Hispanic	1.34 (1.11–1.63)	1.15 (0.94–1.41)

*AA* African American, *BMI* body mass index, *MELD* model for end-stage liver disease

^a^Odds ratios adjusted for age, gender, BMI, diagnosis, MELD score at listing, median household income, and health insurance status

The Cox proportional hazard regression results of the waiting time before access to liver transplantation for both pre- and post-Share 35 periods were presented in Table 3. AA patients had to wait approximately 20% longer on the waiting list before receiving liver transplantation compared to white patients (HR: 0.82, 95% CI, 0.61–0.88), and this situation did not improve after the Share 35 policy (0.81, 0.60–1.10). On the other hand, Hispanic patients experienced a significantly longer waiting time to liver transplantation compared to white patients (0.66, 0.53–0.82), but they trended toward a slight shorter waiting time (0.69, 0.53–0.88) due to the Share 35 policy.

**Table 3:** Cox proportional hazard regression results for the waiting time before liver transplantation among patients registered on waiting list: Pre-Share 35 vs. Post-Share 35 time periods, 2012-2015

	Pre-Share 35 (*n* = 7450)	Post-Share 35 (*n* = 7476)
	Hazard Ratio (95% Confidence Interval)^a^	Hazard Ratio (95% Confidence Interval)^a^
White	1.00 (reference)	1.00 (reference)
AA	0.82 (0.61–1.09)	0.81 (0.60–1.10)
Hispanic	0.66 (0.53–0.82)	0.69 (0.53–0.88)

*AA* African American, *BMI* body mass index, *MELD* model for end-stage liver disease

^a^Hazard ratios adjusted for age, gender, BMI, diagnosis, MELD score at listing, median household income, and health insurance status

## Discussion

In the United States, minority patients account for approximately 30% of all adult liver transplantations performed annually [8]. Racial and ethnic disparities in transplantation have been framed as a combination of barriers in access to care [16]. Overall, the study observed significantly decreased time on waiting list and higher proportion of patients listed with MELD scores over 35 after the Share 35 policy. Subgroups of minority candidates no longer had lower transplant rates than non-Hispanic whites. Hispanics experienced shorter waiting time before the receipt of liver transplantation but still longer than their white counterparts. To the best of knowledge, this study was the first study to explore racial and ethnic inequity in access to liver transplantation for patients with ESLD after the Share 35 policy instituted in June 2013.

Previous disparity-related studies have only stratified on Organ Procurement and Transplant Network (OPTN) regions and some even have not correctly adjusted for geographic or transplant center factors that may affect the receipt of liver transplantation [11, 17, 18]. Patients treated within a hospital or transplant center tend to be more alike, but the likelihood of receiving a liver transplantation varies between transplant centers in different parts of the country and is related to the local availability of deceased organ donors [14]. This violates the heart of classical statistical estimation assumption that treating patients as independent individuals. Geographic variation has also been considered as a threat to delayed access to specialized health care or has been directed to limited local transplant facilities and hepatology expertise [14]. Thus, failure to account for the dependence between individual patients and the transplant centers to which they belong can have profound implications such as falsely narrow confidence intervals and falsely low *p*-values, which means the risk of a false-positive result is increased [19]. Our study went beyond the previous ones that a carefully designed statistical marginal approach with a working independence assumption was incorporated so that each region or transplant center was treated as a cluster in all regression models to account for the impact of geographic variation and transplant center.

For patients who were added to the UNOS waiting list, studies in the pre-Share 35 era found significant racial and ethnic differences in waiting list outcomes, as measured by death prior to transplantation or removal from the waiting list due to being too sick for transplantation [6, 20]. An important finding in our study was the lack of overall disparities in transplant rates between ethnic minorities and non-Hispanic whites, and in fact, there appeared to be a slightly increase in the number of minorities on waiting list and relatively stable percentages of minorities who had access to liver transplantation. AAs and Hispanics did present with more advanced disease at registration in comparison with whites, but equitable transplant rates were observed after accounting for other demographic, clinical and geographic characteristics. This finding was comparable to preliminary results of recent studies after Share 35 policy took effect [11, 21–23]. Therefore, the implementation of Share 35 policy, with its emphasis on reducing mortality on waiting list, did not lead to improved access to liver transplantation so far but eliminated the previously observed racial and ethnic disparity in waiting list.

Another notable finding in our study was that compared to non-Hispanic white patients, AAs and Hispanics still need to wait 20-40% more time before they received liver transplantation, regardless of Share 35 policy. In previous studies, delayed in access to liver transplantation were found likely to result in higher MELD scores, more advanced disease, greater disease-related morbidity, impaired access to quality pre-transplant care, and it may also be associated with worse post-transplant outcomes [6, 8, 16]. One of the possible barriers behind the long waiting time among minorities included the partly shortage of eligible liver donors in the donor pool where the majority of donors are white race [24], since there have already been numerous studies highlighted the adverse impact of donor and recipient race mismatch on post-transplant outcomes among minorities [25–27]. Minority patients therefore tend to face a dilemma situation, that is, either to transplant with a racially mismatched donor in order to shorten their time on waiting list but taking the risk of adverse graft outcomes, or to wait for a racially matched liver at the cost of increased MELD scores. This can be accomplished by encouraging more minority liver donors into the donor pool.

Despite the racial and ethnic disparities alone, socioeconomic status and health insurance may also play critical roles in affecting the access to liver transplantation. According to the National Healthcare Disparities Report, health insurance was the key barrier to healthcare access among AAs and Hispanics [28]. Our study emphasized that AA and Hispanic patients were less likely to have private insurance, but more likely to be insured by Medicaid than white patients. Recent studies have documented a strong association between health insurance status and the likelihood of registered for liver transplantation [11, 29–32]. Insurance may alter a recipient’s choice of a transplant center and increased the risk of receiving liver transplantation with more severe liver disease.

This study has limitations inherent to the retrospective nature and lack of systematic data collection of the UNOS database. Racial and ethnicity data were self-reported, which was prone to misclassification bias. Several studies measuring agreement between self-reported, administrative measures, and phenotype-based information for Medicaid enrollees or patients have shown inconsistency in reporting of race and ethnicity, particularly for persons who self identified as Hispanic having greater odds of being misclassified in administrative data [33–36]. Other races including Asian, American Indian/Alaska Native, Native Hawaiian/Other Pacific Islander, and multi-races were not included in the study due to small numbers of population. Also, health insurance status was the only socioeconomic characteristic available in the UNOS database at individual level that has been considered to be associated with the access to liver transplantation. Characteristic such as income was approximated by median household income in current dollars at census tract level, which may lose individual information. However, due to the size of the database, the analysis of the UNOS is so far the most possible and comprehensive analysis of waiting list today.

## Conclusion

In summary, determining whether racial and ethnic disparities in access to liver transplantation exist is essential. The differences found in our study present challenges that contribute to the health disparities observed in our diverse population. Comparison of the pre- and post-Share 35 periods showed current racial and ethnic variations in access to liver transplantation. The findings of the study may serve as a reminder for the future research of physicians and policy makers who involved in the care of liver transplantation to explore quality improvement and other implementation strategies to monitor and reduce disparities.

## Abbreviations

BMI: Body mass index
ESLD: End stage liver disease
MELD: Model for end-stage liver disease
STAR: The standard transplant analysis and research
UNOS: United network for organ sharing
